# Synthesis of rare earth doped MoS_2_ by the co-pyrolysis of molecular precursors

**DOI:** 10.1038/s41598-026-44301-1

**Published:** 2026-03-19

**Authors:** Ye Cao, Maria Alfredsson, Alan V. Chadwick, Ryan Parmenter, Daniel Dyer, Adam Brookfield, Floriana Tuna, David J. Lewis, David J. Binks

**Affiliations:** 1 Department of Physics and Astronomy, University of Manchester, Manchester, UK; 2 School of Chemistry and Forensic Sciences, University of Kent, Kent, UK; 3 Department of Chemistry, University of Manchester, Manchester, UK; 4 Department of Materials, University of Manchester, Manchester, UK

**Keywords:** Metal-organic complexes, 2D Materials, Rare earths, Chemistry, Materials science

## Abstract

The synthesis of Er- and Nd-doped MoS_2_ powders by the co-pyrolysis of molecular precursors is demonstrated by heating *tetrakis-*(diethyldithiocarbamato) molybdenum(IV) (Mo(DTC)_4_) and *mono-*1,10-phenanthroline *tris*-(diethyl dithiocarbamato) erbium(III) or *mono-*1,10-phenanthroline *tris*-(diethyl dithiocarbamato) neodymium(III) (Ln(DTC)_3_phen, where Ln = Er, Nd) to 500 °C for 1 h. Powder x-ray diffraction and Raman spectroscopy indicated that the resulting powders produced from this reaction are comprised of 0D nanoscale crystallites of the 2 H phase of MoS_2_. Spatial mapping of elements in these samples was performed in the scanning electron microscope at the microscale using energy dispersive X-ray spectroscopic mapping, which showed that the dopants are homogeneously distributed throughout the samples. Synchrotron radiation x-ray absorption measurements tentatively indicate that the dopants are incorporated within the MoS_2_ layers rather than being intercalated between them. Electron paramagnetic resonance spectroscopy and SQUID magnetometry demonstrated that rare earth doping of MoS_2_ significantly enhances the magnetic response of the material. Remarkably, the samples are found to remain paramagnetic down to temperature at least as low as 2 K.

**Supplementary Information:**

The online version contains supplementary material available at 10.1038/s41598-026-44301-1.

## Introduction

The weak van der Waals (vdW) interlayer bonding combined with inert, lone-pair terminated, basal planes in transition metal dichalcogenides (TMDs) allows crystals of atomic thickness to be produced that are thermodynamically stable^1^. The properties of these 2D materials, which have been referred to as ‘inorganic graphene analogues’,^2^ can be tuned via quantum confinement effects by control of their thickness at the nanoscale^3–5^. Indeed, the period 5 and 6 group-VI TMDs, which can be represented by the general formula MX_2_ (where M = Mo, W, and X = S, Se, Te), exhibit thickness-dependent indirect band gaps from near-infrared to the visible up to the bilayer limit, with a switch to direct band gaps in the monolayer limit,^6^ with a concomitant increase in photoluminescence quantum yield^7,8^. The TMDs have large exciton binding energies (0.5-1 eV)^9^ due to the effective dielectric screening ubiquitous in 2D materials so that the constituent electron and hole remain bound to each other even at room temperature, increasing their probability of recombination^10,11^.

The properties of 2D TMDs can be further enhanced by doping with rare earth (RE) ions. This can allow the two-dimensional material to act as a crystalline host for RE ions, which can then produce long-lived emission from the 4f states with energy commensurate with telecomms bands making them well-suited as laser gain media,^12^ and as a platform for quantum technologies, such as single photon sources and quantum memories^13^. RE ions with + 3 charge are electron acceptors in TMDs^14^ and modelling also indicates the RE doping can be used to passivate defect states, reducing the bandgap energy but increasing the optical absorption coefficient^14–16^.

Doping with RE ions that have unpaired electrons in the 4f orbitals has also been used to enhance the magnetic properties of TMDs. Both nanoscale MoS_2_ sheets doped with Dy to a level of 1 at%^17^ and MoS_2_ single crystals doped with Nd to 1 at% result in room temperature ferromagnetism being observed,^18,19^ with the ferromagnetic ordering attributed to coupling between RE ions and defects in the crystalline host, with the ferromagnetism disappearing after annealing for the case of Nd-doped MoS_2_^18^. Doping with RE atoms with unpaired electrons also has the potential to enable low temperature paramagnetism. In this case, materials should be fabricated with their paramagnetic atoms sufficiently well-spaced from one another and from any magnetic defects to suppress their interaction thus avoiding a ferromagnetic transition.

RE-doped 2D TMDs can be fabricated using bottom-up approaches such as pulsed laser deposition (PLD), molecular-beam epitaxy (MBE) and chemical vapor deposition (CVD)^20^. However, commonly used RE precursor materials have high melting points,^21^ making fabrication expensive and control of dopant concentration non-trivial. An alternative synthetic approach is the decomposition of metal-diethyldithiocarbamate (DTC) precursor complexes^22,23^ at relatively low temperatures (~ 500 °C) and ambient pressures, which is an approach previously used to fabricate undoped MoS_2_. This approach benefits from straightforward scalability, requiring only powdered precursors and thermal energy, and can also be employed to produce MoS_2_ doped with a variety of metals. By the co-pyrolysis of two or more precursors in tandem^24^ Mo alloys^25^ and MoS_2_ doped with Cr^22^ have been produced. Recently, this approach has been extended to produce high entropy 2D metal dichalcogenides that act as extremely efficient electrocatalysts for the hydrogen evolution reaction^26^. Importantly, it has been shown that for DTC precursors the metal-to-metal ratio in a mix of precursors is retained in the final solid-state products, thus enabling control of the dopant concentration^27–29^. In theory, this approach could therefore be extended to the use of molecular precursors that encode for the incorporation of RE ions into the MoS_2_ host.

In this article, the objective is to demonstrate the synthesis of RE-doped MoS_2_ powders by the co-pyrolysis of metal organic precursors for the first time, and this is done using Mo-DTC with either Er- or Nd- DTC. These precursors were selected for this study because they have already been shown^30^ to decompose to the corresponding lanthanide (Er or Nd) sulfide, suggesting that their co-pyrolysis with Mo-DTC would lead to lanthanide-doped (Er or Nd) MoS_2_. The products were analyzed by energy dispersive x-ray (EDX) spectroscopy, scanning electron microscopy (SEM), x-ray diffraction (XRD) measurements, and Raman spectroscopies to confirm Er or Nd doping. X-ray absorption fine structure (XAFS) analysis was used to identify the position of any Er or Nd dopants in the MoS_2_ lattice, and the mode of their incorporation e.g. bonding in-plane or intercalated between crystalline layers. Finally, the temperature-dependent magnetic properties of samples from the reactions were compared using a SQUID magnetometer and electron paramagnetic resonance (EPR) spectroscopy. It was found that the magnetic response was significantly increased by both Er and Nd doping and, notably, the material remained paramagnetic down even at 2 K, the lowest temperature studied.

## Results

### Assessing precursor suitability for co-pyrolysis

**Fig. 1:**
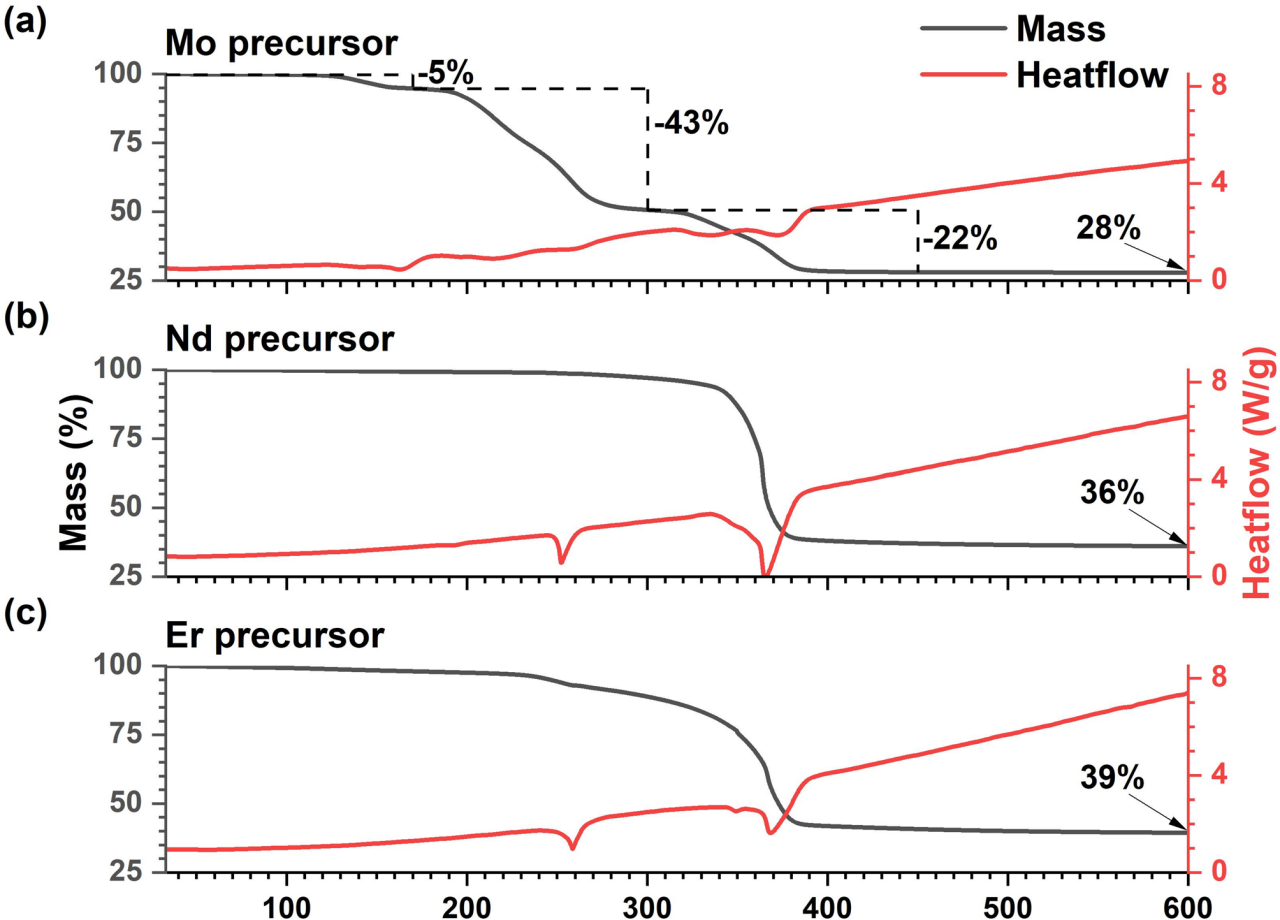
TGA (black) and DSC (red) for the **a**, Mo, **b**, Nd and **c**, Er complexes. The three-step decomposition has been indicated for the Mo precursor. The temperature was held constant after reaching 600 °C.

The thermogravimetric analysis (TGA) and differential scanning calorimeter (DSC) data for Mo(DTC)_4_ is given in Fig. 1a. The profile recorded is in good agreement with a previous report with the thermal decomposition of Mo(DTC)_4_ being a three-step process^31^. The first step is attributed to the loss of ethylene; the second corresponds to the loss of two DTC molecules per complex; and the third step to the loss of the final DTC molecules from each complex. The TGA and DSC data for the Nd and Er samples (Fig. 1b and c, respectively) are also similar to that observed in previous work^23,30^ indicating that for these molecules, in contrast to Mo(DTC)_4_, thermal decomposition is a two-step process, with the first step attributed to loss of the phen and the second to the loss of the DTC. These TGA and DSC data show, crucially, in each case the final decomposition step to the sulfide occurs over the same temperature range of 360 °C to 380 °C indicating that it is dictated by the ligand structure rather than the metal. The co-incidence of the final decomposition temperature ranges enables the synthesis of Ln-doped (Er or Nd) MoS_2_ by heating a mixture of precursors.

### Characterization of the rare earth doped MoS_2_ powders

**Fig. 2:**
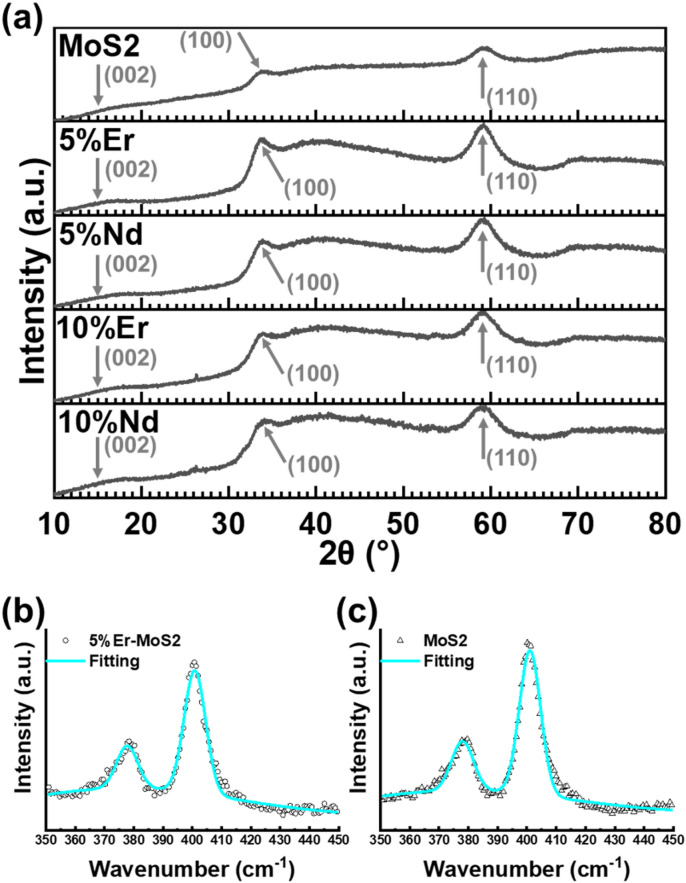
**a**, Comparison of XRD spectra of the undoped, 5% Er, 5% Nd, 10% Er, and 10% Nd doped MoS_2_ samples; the arrows indicate the position of the (002), (100) and (110) peaks at ~ 15º, 33.7º and ~ 59.2º, respectively. **b** and **c**, Raman shift spectra for 5% Er doped and undoped MoS_2_ on laser excitation at 488 nm.

Figure 2a compares the XRD spectra of the undoped and Ln-doped (Er or Nd) MoS_2_ samples. Reflections evident at 2θ = 33.7° and 59.2° correspond to the (100) and (110) Bragg planes of hexagonal 2 H-MoS_2_^32^. Broad features are also discernible (particularly after background subtraction – see Fig. S2 in the Supporting Information for example) centered at 2θ = 15°, 37° and 70°, corresponding to the (002), (103) and (201) planes of MoS_2_^32^. The width of the peaks at 33.7° and 59.2° was determined from fitting after baseline subtraction, (see example in Fig. S2 and Table S2), and used to estimate the crystallite size via the Scherrer equation, yielding average sizes of 3.7$\pm$0.1 nm and 2.6 $\pm$0.1 nm for the (100) and (110) crystal planes, respectively. No significant difference in peak position is evident between the doped and undoped samples, suggesting that doping does not introduce significant strain to the crystal lattice. The extreme broadening of the (002) reflection at 2θ ~ 15°, which corresponds to the interlayer distance in bulk samples, has been observed previously from pyrolysis of the Mo-DTC precursor^25,33^ and is characteristic of ultrathin sheets of MoS_2_^34^. Note that these figures are potentially not absolute values, as instrumental line broadening (0.09° full width at half maximum) was not corrected for, but are still useful to determine the approximate crystallite domain size, and relative sizes of the crystallites in samples compared with each other.

Figure 2b and c compare the Raman spectra for the 5% Er doped and undoped MoS_2_ samples; spectra for the other samples are given in the Supporting Information in Fig. S2 with the peak positions and widths given in Table S3. For each sample, peaks are found at 378 and 401 cm^− 1^ (except for the 5% Nd-doped sample for which the peaks are red-shifted by 1 cm^− 1^ and 0.5 cm^− 1^, respectively, ) and correspond to the in-plane E^1^_2g_ mode and the out-of-plane A_1g_ mode of MoS_2_^35^. Several factors are known to affect the position of these peaks: the E^1^_2g_ phonon is redshifted with increasing strain whilst the A_1g_ phonon is redshifted by increasing free electron concentration^36–39^. Quantum confinement also affects the position relative to each other^40^. The shift of one peak relative to the other can also be indicative of the location of doping within the lattice^41^. The absence of a significant difference in peak positions between the doped and undoped samples indicates that the doping with Er or Nd up to 10% does not have an appreciable effect on strain or carrier concentration in these samples. MoS_2_ is commonly found to be n-type due to S vacancies acting as donors and so this lack of a doping related shift, despite the Er and Nd dopants being expected to act as acceptors, may be explained by a large background carrier concentration. The difference in wavenumber between the E^1^_2g_ and A_1g_ peaks has been previously shown to be determined by the number of monolayers, with the value of 23 cm^− 1^ observed here indicating a thickness of 2 to 3 monolayers^35^. The wavenumber difference between the E^1^_2g_ and A_1g_ peaks has also been shown to be weakly dependent on doping level for rhenium-doped MoS_2_^42^ but no similar shift is observed here between the doped and undoped samples, within the resolution of the measurement.

**Fig. 3:**
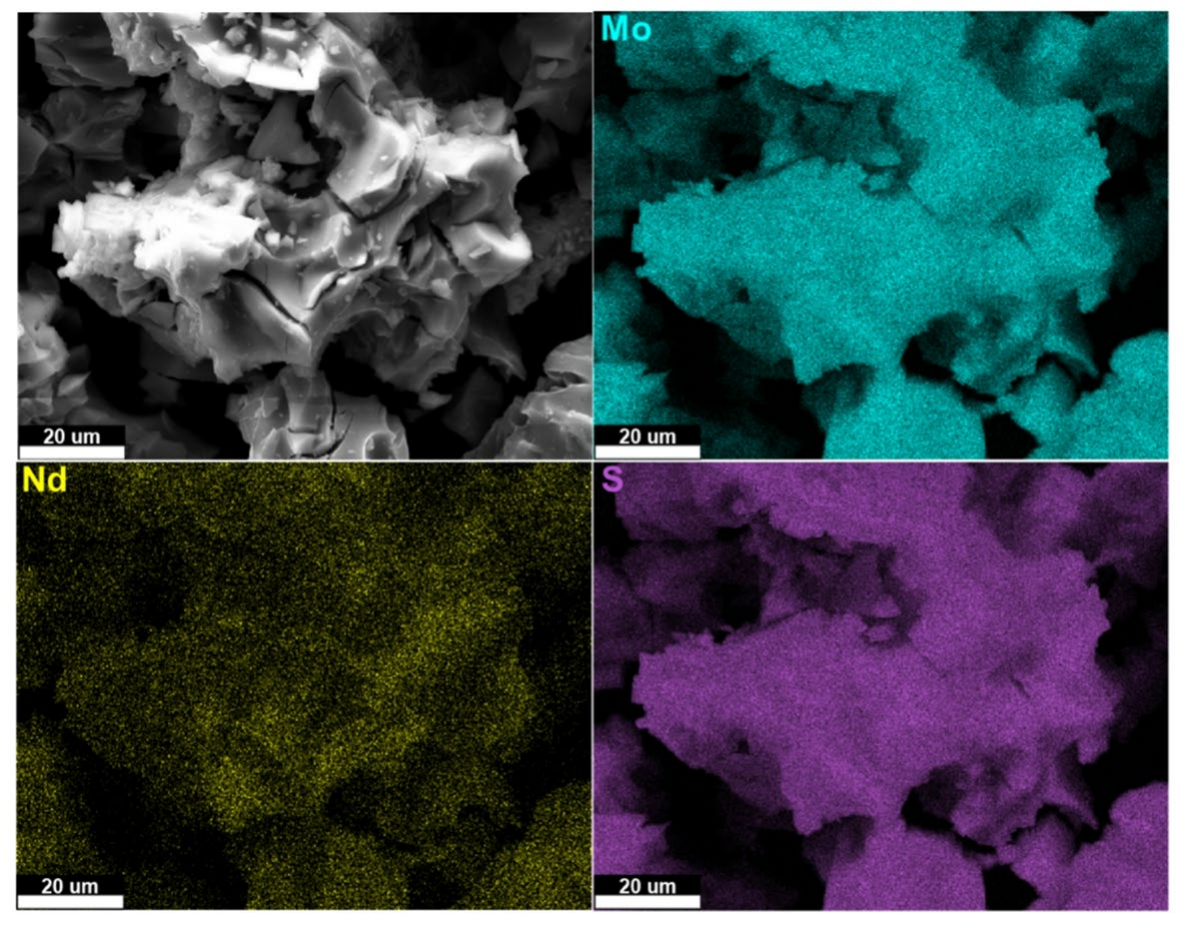
SEM (secondary electron) imaging of 5% Nd doped MoS_2_ samples (top left) and EDX mapping of the distribution of Mo (L α, 2.293 keV, top right), S (K α, 2.307 keV, bottom right), and Nd (L α, 5.229 keV, bottom left). Imaging was performed at an accelerating voltage of 30 kV.

Representative SEM images and EDX maps of the 5 at% Nd doped sample are shown in Fig. 3, with similar images for the other samples given in the Supporting Information (Fig. S3). The images in Figs. 3 and S3a indicate that there is a largely homogeneous distribution of Mo, S, and the Nd dopant; similarly, the images in Figures S3b and c show that there is a largely homogeneous distribution of the Er dopant. A similar homogeneous distribution was achieved previously using the thermolysis of molecular precursors to synthesize MoS_2_ doped by chromium^22^ and rhenium^42^. These results indicate that the use of Er or Nd containing metal-organic precursors, the thermal decomposition temperature of which is largely determined by the ligands rather than the metal species, enables the simultaneous availability of both dopant atoms and the metal atoms corresponding to the host materials and thereby effective doping with rare earths, as well as transition metals. The relative atomic abundances of Mo and the Er or Nd dopants determined from these EDX images are given in Table S4 of the Supporting Information and were used to estimate the doping fraction achieved. These estimates were all within 10% of the target doping fractions apart from the nominally 10% Er-doped sample, for which the doping fraction estimated from the EDX data was 7.8%.

**Fig. 4:**
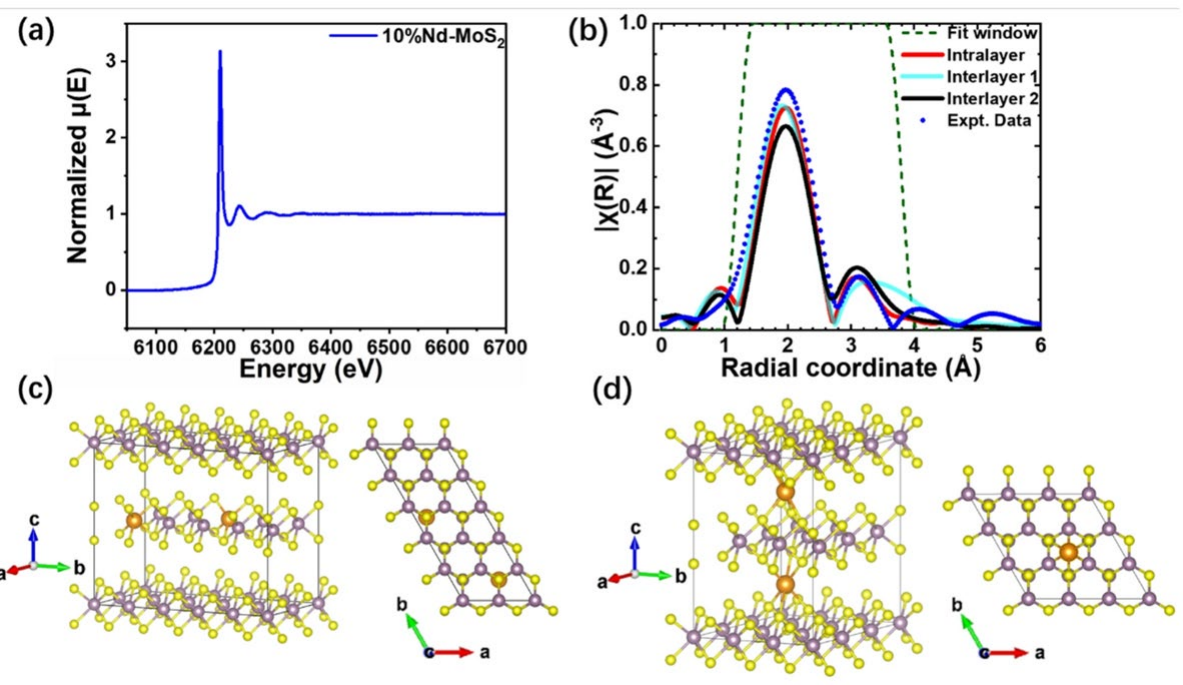
Shows (a) the normalized absorption spectrum *µ*(*E*) for 10% Nd-doped MoS_2_ sample; (b) the Fourier transform of the absorption spectrum and fitting in *R* space (without phase correction), with *k* weight of 2. Fourier transform is within 2.0–6.5 Å^−1^ in *k*-space applying a Hanning window function rolled off over 1 Å^−1^. The fitting over *R*, from 1.2 to 3.7 Å with a Hanning window rolled off over 0.5 Å, is compared for the intralayer model and both interlayer models. (c) and (d) show visualizations of intralayer and interlayer (geometry 1 - trigonal prism of nearest S) incorporation of the Nd dopants considered for comparison with experiment. (n.b. the yellow, purple and orange balls represent S, Mo and RE atoms respectively).

XAFS spectroscopy has been used previously to investigate how various dopants are incorporated into MoS_2_ samples including Co^43^, Pt^44^ and Pd^45^. Hence, the 10% Er- and Nd-doped samples were investigated by XAFS spectroscopy using synchrotron emission as the tunable radiation source. Figure 4a shows the normalized x-ray absorption coefficient spectrum, *µ*(*E*), for the 10% Nd-doped sample in the region of the L3 edge of Nd; similar data for the 10% Er-doped sample is given in the Supporting Information (Fig. S4). The oscillations in the absorption spectrum (merged from three scans) seen for x-ray energies up to ~ 100 eV greater than the absorption edge are due to scattering of the photoelectron from neighboring atoms. The k-space fitting results to these oscillations and associated residuals are shown in Fig. S5 of the Supporting Information. These data were used to compute the XAFS fine-structure function, |*χ*(*R*)|, where $R$ is the radial coordinate (without phase correction, also see Methods for details). This function is shown in Fig. 4b and is dominated by a broad peak centered at $R=$2.0 Å and spanning the range $R=$ 0.8–2.8 Å, which corresponds to scattering by the nearest neighbor atoms to the Nd. Smaller peaks are evident at larger $R$ values, which progressively diminish in amplitude with increasing $R$; these correspond to scattering by more distant atoms. This data was compared to two models: intralayer incorporation, in which the Nd substitutes for Mo in the lattice, and interlayer incorporation, in which the Nd is sandwiched between two MoS_2_ layers. Both models are based on an adapted 2 H- MoS_2_ unit cell^46^. The intralayer case was derived from a 2 × 5 × 2 supercell, substituting Nd for two Mo atoms and losing one S atom to balance charge to yield Mo_18_Nd_2_S_39_, and the interlayer model was adapted from a 3 × 3 × 2 MoS_2_ supercell, inserting two Nd atoms between layers to yield Mo_18_Nd_2_S_36_. For the interlayer model, two possible geometries were considered: (1) the Nd is located in the body center of a triangular prism consisting of the nearest six sulfur atoms, and (2) the Nd bonds with 4 sulfur atoms to form a tetrahedral structure, assuming each bond length is the same to simplify modeling. Visualizations of the intralayer model and the interlayer model with geometry 1 are shown in Fig. 4c and d, respectively; a visualization of the interlayer model with geometry 2 is shown in Fig. S6. These models and geometries were used as the basis of a fit to the |*χ*(*R*)| data for both the Nd- and Er-doped samples. Figure 4b compares the fits for each model for the Nd-doped sample. The fit to the intralayer model most closely matches both the amplitude and width of the peaks of the XAFS data within the fitting window. In contrast, for the interlayer models, the width of the fitted peak centred at 3.1 Å is significantly broader than the experimental peak for geometry 1, whereas the amplitudes of the fitted peaks for geometry 2 correspond less well to the experimental amplitudes than do either of the other models. The fits to the |*χ*(*R*)| data for the Er-doped sample are given in Fig. S4. Similar observations can be made about the fits in this case: the intralayer model best describes the experimental data with interlayer model geometry 1 not matching the data well for *R* values from about 2.5 to 4.2 Å and interlayer model geometry 2 corresponding less well to the peak amplitudes than the other models. The parameters resulting from all fits are also given in the Supporting Information (Tables S5-S8). The XAFS data thus tentatively indicate that both the Er and Nd dopants most likely substitute for Mo atoms within each layer, rather than being trapped between layers. Such intralayer incorporation of the Nd is also consistent with the much lower formation energy calculated for Nd substituting on the Mo site (< 0.2 eV) compared to the incorporation of Nd between layers (> 2 eV)^18^. Similar studies on doping MoS_2_ with Co^43^, Pt^44^ and Pd^45^ have also found that the corresponding XAFS data is consistent with intralayer rather than interlayer incorporation. Moreover, the studies on Pt and Pd doping also concluded that these dopants substituted for Mo atoms within layers, consistent with the intralayer model used here to describe Ln (Er or Nd) -doped MoS_2_.

### Functional properties of rare earth doped MoS_2_ powders

**Fig. 5:**
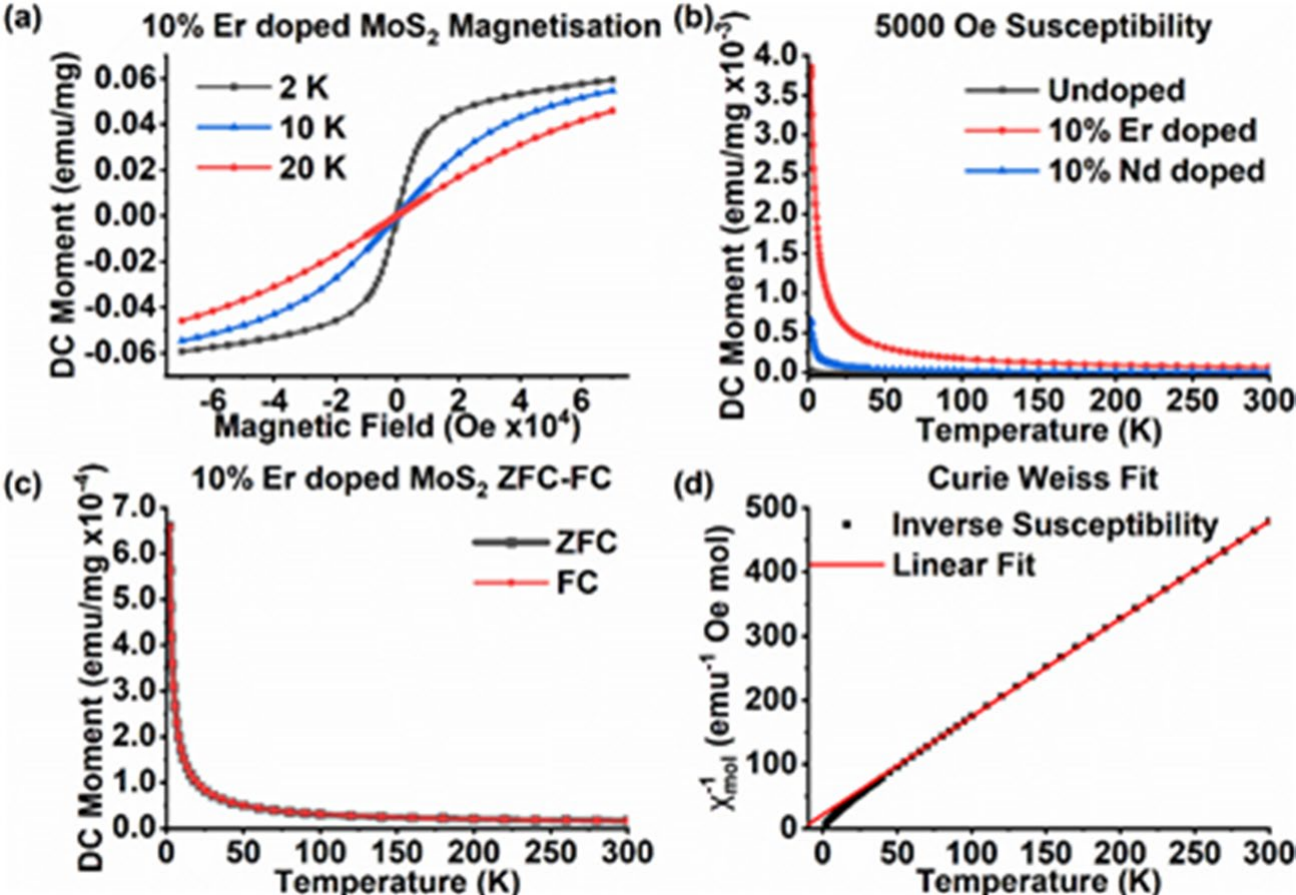
**a**, Magnetic response of the 10% Er doped MoS_2_ sample at low temperature. **b**, Comparison of the temperature dependence of magnetization for 10% Er-,10% Nd-doped and undoped MoS_2_ at 5000 Oe. **c**, Field cooled (FC) and zero field cooled (ZFC) magnetization response at 100 Oe for the 10% Er-doped sample. d) Temperature dependence of the inverse molar susceptibility, ${\chi}_{mol}^{-1}$; the fit shown is the Curie-Weiss law for temperatures > 50 K.

Photoluminescence (PL) emission was found to be absent for the Nd-doped samples and very weak for the Er-doped samples, even at low temperature. It has previously been shown^47^ that the PL quantum yield of MoS_2_ depends strongly on the number of monolayers, falling from a few percent for a single monolayer to less than 10^− 4^ for two or more monolayers as the bandgap goes from direct to indirect. The weak or absent PL observed here is thus consistent with the thickness of 2–3 monolayers indicated by the separation of the E^1^_2g_ and A_1g_ Raman peaks described above. Nevertheless, PL peaks corresponding to transitions in Er^3+^ were identified (see Supporting Information Fig. S7). In contrast, RE-doping had a significant effect on the magnetic properties of MoS_2_. The magnetic moment per mg in the 10% Er-doped MoS_2_ sample produced by an applied magnetic field at low temperature is shown in Fig. 5a. A clear paramagnetic response is seen that approaches saturation at the highest fields at 2 K, indicating almost complete alignment of the spins. Similar data for the 10% Nd-doped sample is given in Supporting Information (Fig. S8). The temperature-dependent moment per mg of the 10% Er- and 10% Nd-doped and undoped samples are compared in Fig. 5b and shows that this paramagnetic response is largely due to the RE-doping with the maximum response of the Er-doped sample nearly 4 times greater than that of the Nd-doped sample. This difference in response can largely be attributed to the larger magnetic moment of Er ions compared to Nd ions, as discussed below. The temperature-dependence of the magnetic moment when the doped samples are cooled under a 100 Oe field and under zero field are compared in Figs. 5c and S8c. There is no discernible difference between the cooling curves indicating that this no hysteresis across this temperature range and confirming that there is no ordering of the spins down to 2 K, the lowest temperature accessible in this study, i.e. the material remains paramagnetic even at very low temperatures. The paramagnetism seen here contrasts with the ferromagnetism of the Dy-doped MoS_2_ and the Nd implanted MoS_2_ reported previously,^17,18^ despite the higher doping level in our case. The temperature dependence of the inverse of molar susceptibility, ${\chi}_{mol}^{-1}$ for the 10% Er-doped MoS_2_ sample is shown in Fig. 5d, with the corresponding data for the 10% Nd-doped MoS_2_ sample shown in Fig. S8d. Down to a temperature, $T$, of at least 200 K rare earth magnets typically obey the Curie-Weiss law i.e. ${\chi}_{mol}^{-1}={C}^{-1}\left(T-{T}_{CW}\right)$ where $C$ is the Curie constant and ${T}_{CW}$ is the Curie-Weiss temperature^48^. Here, the response of the Er-doped MoS_2_ is well described by the Curie Weiss law down to 50 K, with the deviation at lower temperatures due to thermal depopulation of the crystal field levels^48^. Fitting to the data for temperatures > 50 K yields a Curie constant of $C$ = 8±1 emu K mol^[−1^, which corresponds to an effective magnetic moment of µ_eff_ = (8 ± 1)µ_B_, consistent with the free ion value of 9.5 µ_B_^48^. In contrast, the data for Nd-doped MoS_2_ deviates from linearity even for temperatures > 200 K. This behaviour indicates that there is a temperature independent component to the susceptibility^48^. Nevertheless, fitting the data for > 200 K with a version of the Curie-Weiss law modified to include such a component yields $C$ = 1.2 ± 0.1 emu K mol^− 1^ and µ_eff_ = (3.1 ± 0.2) µ_B_ for the Nd-doped sample, with the latter being consistent with the free ion value of 3.4 µ_B_^48^. As noted in the Introduction, the ferromagnetic ordering observed previously was attributed to coupling between the RE dopants and defects^17,18^ suggesting a significantly lower concentration of magnetic defects in our Er- or Nd-doped samples. EPR spectroscopy was thus used to investigate the paramagnetism of the doped samples in more detail.

**Fig. 6:**
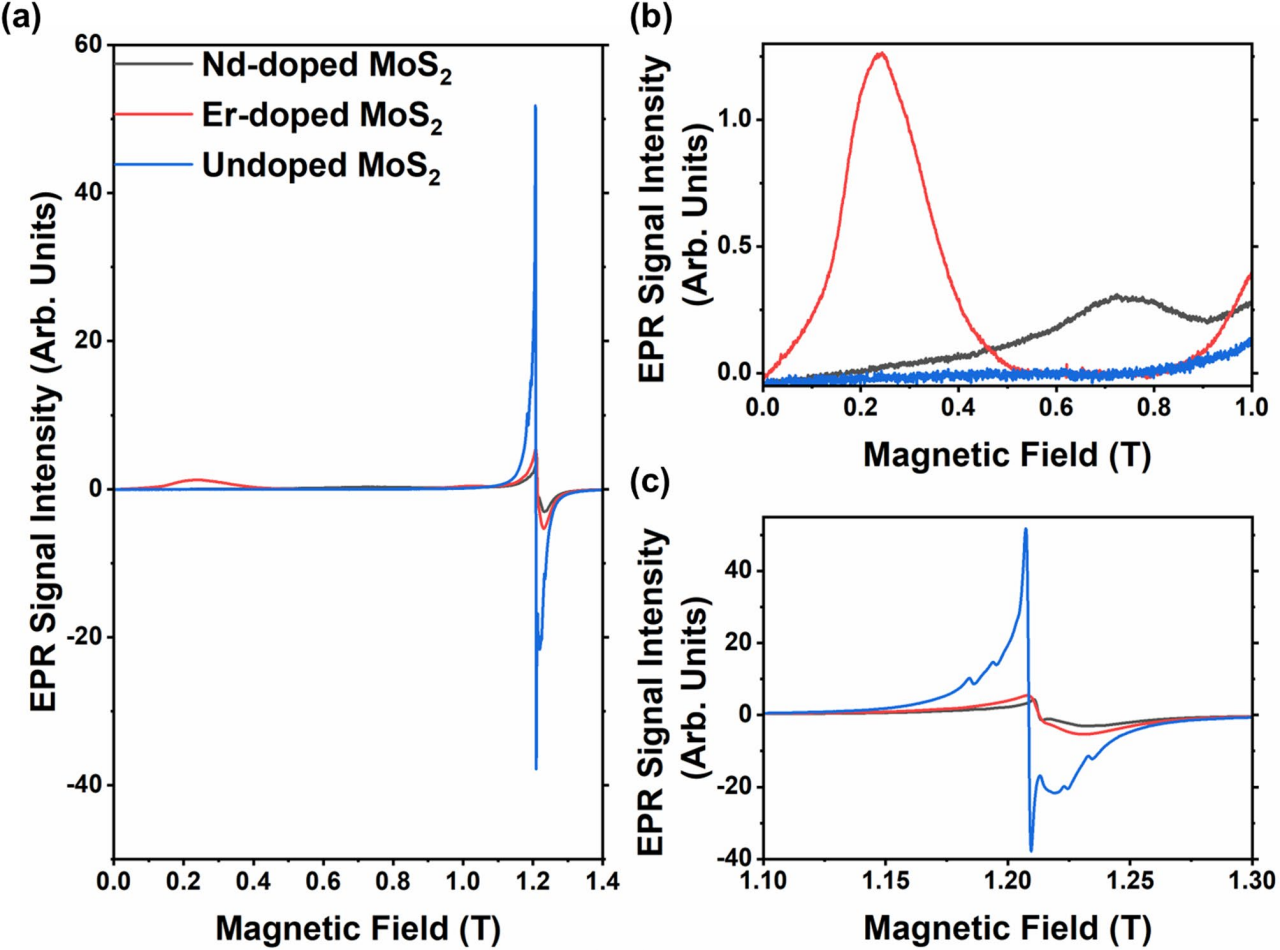
a. EPR spectra of equal masses of the undoped, 10% Er -doped and 10% Nd-doped MoS_2_ samples measured in the Q-band (34 GHz) at <8 K. b) and c) show details of the features for magnetic fields up to 1 T and around 1.21 T, respectively.

Figure 6 compares the low temperature EPR spectrum for the undoped, 10% Er- and 10% Nd-doped MoS2 doped. At low field (0.1–0.4 T, g$\approx$9.7), a broad peak is seen in the spectrum for the Er-doped samples that is absent in the undoped spectrum (see Fig. 6b). A similar feature has been reported previously in the EPR spectrum for Er-doped oxyfluoride glasses^49^. Figure 6b also shows the spectra for the Nd-doped sample which contains a broad, 0.2T wide feature centered at about 0.75 T (g$\approx$3.2) that is not seen in the undoped spectrum and so is attributed to Nd^3+^. Centered around 1.21 T (g$\approx$2.007) in the spectrum for the undoped samples is a sharp feature (linewidth = 0.002 T), close inspection of which (see Fig. 6c) reveals fine structure. Previous EPR studies of undoped MoS_2_ have reported a paramagnetic response that was suppressed by annealing under argon and so was attributed to defects such as adsorbed oxygen species, sulfur vacancies, thio-, and oxo-Mo^5+^^50^. The fine structure evident in the EPR peak around 1.2 T for the undoped MoS_2_ is attributed to hyperfine coupling with the 5/2 nuclear spin of the Mo-96 and Mo-97 isotopes (joint natural abundance of 25.5%). In contrast, the only stable isotope of sulfur with a non-zero spin is S-33, which has a very low natural abundance (0.76%) and a nuclear spin of 3/2 and so cannot account for the number of fine structure features observable. A similar feature is also seen in the spectra for both the Er- and Nd-doped samples but with significantly reduced amplitude, consistent with the defect passivating effect of RE dopants predicted theoretically^14,50^. In summary, these data thus suggest that the low temperature paramagnetism that is produced in our MoS_2_ samples by doping is due to both inherent magnetic properties of the Er or Nd dopants and their passivating effects on magnetic defects.

## Discussion

The results presented above demonstrate that the co-pyrolysis of metal-diethyldithiocarbamate precursor complexes enables the synthesis of MoS_2_ nano-crystallites doped with either Er or Nd. Diethyldithiocarbamate precursor complexes also exist for several other lanthanides (Ln = La, Ce, Pr, Sm, Gd, Dy and Ho) that undergo pyrolysis to the sulfide Ln_2_O_3_ at a similar temperature to the Er and Nd complexes studied here^30^. This suggests that the co-pyrolysis method used in this investigation could be extended to dope MoS_2_ with these other rare earths.

The Er- and Nd-doping demonstrated here significantly enhances the magnetic response of the MoS_2_, which remains paramagnetic to temperatures at least as low as 2 K. This suggests that these materials may be able to be used to achieve temperatures lower than this by exploiting adiabatic demagnetization; in this technique, magnetizing a paramagnetic material and then removing the applied field under adiabatic conditions results in cooling as the unpaired spins relax from their low entropy configuration^51^. However, significant further investigation is required before the potential of Er or Nd-doped MoS_2_ for this application can be confirmed.

## Conclusions

This work demonstrates that the co-pyrolysis of metal organic complexes can be used to produce powders of Er- or Nd-doped hexagonal phase MoS_2_. These powders are composed of nano-crystallites of a few nm in size and can be doped up to at least 10% with rare earths. We show, using synchrotron XAFS, that the Er or Nd ions are most likely incorporated within the S-Mo-S crystalline layers and not intercalated within the van der Waals galleries between layers. This doping significantly enhances the magnetic response of the MoS_2_, which remains paramagnetic down to temperatures at least as low as 2 K. This low temperature paramagnetism is a notable result considering that previous work on ~ 1% RE-doped MoS_2_ found that the samples were ferromagnetic even at room temperature. The low temperature paramagnetism observed here is attributed, supported by EPR measurements, to the passivation of defects that would otherwise couple to the dopants resulting in magnetic ordering. Weak photoluminescence was also observed for the Er-doped sample.

## Methods

### Characterisation of molecular precursors

An extended mass range electrospray mass spectrometer (Thermo Orbitrap Exactive Plus) and time-of-flight matrix assisted laser desorption ionisation (TOF-MALDI) mass spectrometer (Shimadzu Biotech Axima Confidence) were used for mass spectrometry of the Mo and RE precursors, respectively. A combined thermogravimetric analyzer and differential scanning calorimeter (Mettler-Toledo) were used for TGA and DSC analysis of the precursors under nitrogen at ramping rate of 10 °C/min up to a maximum temperature of 600 °C. CHN compositional analysis was performed with a Thermo Scientific Flash 2000‌ Smart elemental analyzer. FTIR spectra of the precursors were recorded using a Bruker Alpha FTIR spectrometer.

### Reagents

All chemicals were purchased from Sigma–Aldrich unless specified. Tetraethylthiuram disulfide (≥ 97.0%), molybdenum(0) hexacarbonyl (98.0%), acetone (≥ 99.0%), pentane (≥ 99.0%), sodium diethyldithiocarbamate trihydrate (≥ 98.0%), 1,10-phenanthroline (≥ 99.0%), erbium(III) nitrate pentahydrate (99.9%), neodymium(III) nitrate hexahydrate (9.9%), methanol (≥ 99.5%), acetonitrile (99.9%), hexane (≥ 97.0%), dichloromethane (≥ 99.0%) were used without further purification.

### *tetrakis-*(diethyldithiocarbamato) molybdenum(IV) (Mo(DTC)_4_)

Mo(DTC)_4_ was synthesized in a Schlenk line system under a high-purity nitrogen atmosphere using a procedure detailed previously^22^. First, Mo(CO)_6_ (1.0 g, 3.8 mmol) and tetraethylthiuram disulfide (2.3 g, 7.6 mmol) were dissolved in degassed acetone (35 mL, N_2_ sparge) and heated at reflux for 1.5 h, after which was cooled to room temperature. The solid product was isolated as deep purple-colored crystals by suction filtration, washed with pentane (3 × 20 mL, add by pipette), and then dried in suction filtration at room temperature. Mass spec + m/z: 690 {M + H}^+^. FT-IR (solid) νmax/cm–1:. Anal. Calcd for Mo(DTC)_4_: C, 34.9%; H 5.9%; N, 8.1%. Found: C, 34.6%; H, 5.9%; N, 7.7%.

### *mono-*1,10-phenanthroline *tris*-(diethyl dithiocarbamato) lanthanide (III) complexes (Ln(DTC)_3_phen, where Ln = Er, Nd)

The synthesis of Ln(DTC)_3_phen (Ln = Er or Nd), was undertaken in ambient atmosphere, and has been described previously^23^. NaDTC·3H_2_O (0.68 g, 3 mmol) and 1,10-phenanthroline (0.18 g, 1 mmol) were added to a mixture of methanol (25 mL) and acetonitrile (10 mL) and heated at 70 °C to dissolve the solids. Lanthanide nitrate (0.44 g, 1 mmol) in methanol (5 mL) was added dropwise and a colored precipitate was produced. The mixture was refrigerated at 0–5° C for 1 h to improve the yield of the crystallization. The solid product was dumped into suction filter together with residuals washed by extra acetonitrile (2 volumes of pipette), isolated by suction filtration, and then washed by hexane (about 50 ml, add by pipette). Noticeably, too much acetonitrile may dissolve considerable product during washing. Until all the solvent was drained, the products were contained in vials filled with argon and stored in a refrigerator when not in use. Mass spec + m/z: 620 {Nd(DTC)_2_phen}+, 644. {Er(DTC)_2_phen}+, 800 {Nd(DTC)_2_(phen)_2_ }+, and {Er(DTC)_2_(phen)_2_ }+. FT-IR (solid) νmax/cm–1: 3000 (w), 728,(m) 842 (m), 1417 (m) and 1516 (m) for both Er(DTC)_3_phen and Nd(DTC)_3_phen. Anal. Calcd for Nd(DTC)_3_Phen: C, 42.2%; H 5.0%; N, 9.1%. Found: C, 41.8%; H, 4.9%; N, 9.0%. Anal. Calcd for Er(DTC)_3_Phen: C, 40.9%; H 4.8%; N, 8.8%. Found: C, 39.6%; H, 4.9%; N, 8.6%.

### Synthesis of Ln (Er or Nd)-doped MoS_2_ powders by co-pyrolysis of molecular precursors

RE-doped MoS_2_ powders were synthesized by co-pyrolysis of the Mo and Ln (Er or Nd) complexes in a high-purity argon atmosphere. Mo(DTC)_4_ (0.172 g, 0.25 mmol) powder was mixed with Ln(DTC)_3_phen (Ln = Er or Nd) powder at 0 at%, 5 at% (mass(Ln precursor) = 0.1 g, 1.3 ⅹ 10^− 2^ mmol), and 10 at% (mass(Ln precursor) = 0.2 g, 2.8 ⅹ 10^− 2^ mmol) ratios (n.b. undoped MoS_2_ powders were produced by using the Mo complex powder only, decomposing in furnace without pre-dissolving in dichloromethane). The mixed complexes were dissolved in dichloromethane (20 mL) and shaken up. This solution was then heated to 40 °C on hotplate until all the dichloromethane evaporated. The residual solid was transferred to a ceramic vessel and placed inside a quartz tube in the middle of a tube furnace (Carbolite Gero), and the tube flushed for 20 min with Ar (200 cm^3^/min) after which the Ar flow was reduced (50–100 cm^3^/min). The furnace was set to 500 °C with 10 °C/min ramp rate and was held at this temperature for 1 h. The furnace was allowed to cool to room temperature naturally, and the products of the co-pyrolysis reaction were collected from the ceramic boat.

### Structural characterization of Ln (Er or Nd)-MoS_2_

Powder XRD spectra for the doped and undoped MoS_2_ powders were taken using a PANalytical (Phillips) X’Pert Pro in Bragg-Brentano 2θ configuration (Cu Kα1 = 1.540598 Å, Kα2 = 1.544426 Å, Kα ratio 0.5, Kαav = 1.541874 Å). The center positions and widths of the peaks in the spectra, and the associated uncertainties, were found by fitting Gaussian functions to them. The instrumental linewidth was estimated using a silicon reference standard and found to be 0.09° (full width at half maximum). A FEI Quanta 200 (E) SEM equipped with EDAX OCTANE 70 mm^2^ SSD detector was used to image powders and obtain spatially resolved EDX spectroscopic maps. A Horiba LabRAM Evolution HR with a resolution of 0.7 cm^− 1^, equipped with a 50 × objective, was used to obtain Raman spectra, with the laser excitation wavelength being 488 nm.

### Optical characterization of Ln (Er or Nd)-MoS_2_

Photoluminescence spectra were acquired under excitation by 100 fs pulses from a mode-locked Ti: sapphire laser frequency-doubled to a wavelength of 400 nm. The laser delivered 40.2 pJ per pulse with an estimated spot size of 12.7 μm at the sample. The sample was cooled to14 K in a closed cycle He cryostat, with the sample emission collected and focused into a SPEX 1702/04 spectrometer and detected by a photomultiplier tube. Lock-in detection was performed by a Stanford Instrument lock-in amplifier to improve the signal-to-noise ratio, with a mechanical chopper within the excitation path acting as the reference signal.

### X-ray absorption spectroscopy of Ln (Er or Nd)-MoS_2_

Samples were prepared for X-ray absorption spectroscopy (XAS) by mixing 24 wt% of the Er- and Nd-doped MoS_2_ powders with cellulose (20 μm microcrystalline), grinding into a fine powder and pressing into 7 mm diameter pellets with a thickness of ~1 mm. XAS was obtained on beamline B18 of the Diamond Synchrotron (Oxfordshire, UK),^52^ collecting data as quick XAFS in both transmission and fluorescence mode. For the transmission mode set-up, a third ion chamber is used to collect transmission spectra from a reference metal foil for energy calibration. In this study Ni and Cr foils were used as reference foils for Er and Nd, respectively. The L3 absorption edge for each of the REs was used in this study. The monochromator used in all measurements is the Si(111), together with the Pt 3rd harmonic mirror. XAFS measurements were collected over 8.15–9.25 keV for the Er L3-edge and 6.00-6.72 keV for the Nd L3-edge. These energy intervals correspond to x-ray wavenumber ranges, $\Delta k$, of 15.3 Å^−1^ and 11.4 Å^−1^, respectively. $\Delta k$ determines the spatial resolution as $\varDelta\mathrm{R}\approx{\uppi}/2\varDelta\mathrm{k}$^53^; for the Er and Nd data this yields $\varDelta\mathrm{R}\approx$0.10 Å and 0.14 Å, respectively. The energy range of the spectra covered both the near-edge region and the extended fine structure region. All analysis was done on the k^2^-weighted XAS. A smoothed background was subtracted from the measured x-ray absorption coefficient spectra, *µ*(*E*), which was then normalized to the absorption edge step; the Fourier transform of this yielded |*χ*(*R*)|, where *R* is radial distance from the RE atom absorbing the x-ray. This was modelled using the Athena and Artemis software packages, the latter including the use of the FEFF code for the calculation of phase shifts and effective scattering amplitudes^53^. The XAFS fitting used starting structural models produced from published crystal structures/theoretically generated CIF-file to refine interatomic distances, coordination numbers and Debye-Waller factors. Full energy ranges were used in the fitting. The EPR instrument used was the Bruker EMXPlus Spectrometer, operating in the Q-band (34 GHz) with magnetic fields up to 1.4 T, respectively. A SQUID magnetometer (Quantum Design MPMS3) was used to determine the temperature dependent magnetization of the samples.

## Supplementary Information

Below is the link to the electronic supplementary material.

Supplementary Material 1

Supplementary Material 2

## Data Availability

The data underlying this study are openly available in the University of Manchester Repository at [https://doi.org/10.48420/29931842](https:/doi.org/10.48420/29931842) .
