# Supplementary material for: Synthesis of rare earth doped MoS2 by the co-pyrolysis of molecular precursors
Source: Sci Rep. 2026 Mar 19;16:14252. doi: 10.1038/s41598-026-44301-1 (PMC13139367; doi:10.1038/s41598-026-44301-1)
Supplement: Supplementary file 1 — Supplementary Material 1 [file 41598_2026_44301_MOESM1_ESM.docx]

Supporting Information for:

Synthesis of rare earth doped MoS_2_ by the co-pyrolysis of molecular precursors

*Ye Cao^1^, Maria Alfredsson^2^, Alan V. Chadwick^2^, Ryan Parmenter^2^, Daniel Dyer^1^, Adam Brookfield^3^, Floriana Tuna^3^, David Lewis^4^ and David J. Binks^1,^**

1. Department of Physics and Astronomy, University of Manchester, Manchester, UK
2. School of Chemistry and Forensic Sciences, University of Kent, Kent, UK.
3. Department of Chemistry, University of Manchester, Manchester, UK
4. Department of Materials, University of Manchester, Manchester, UK


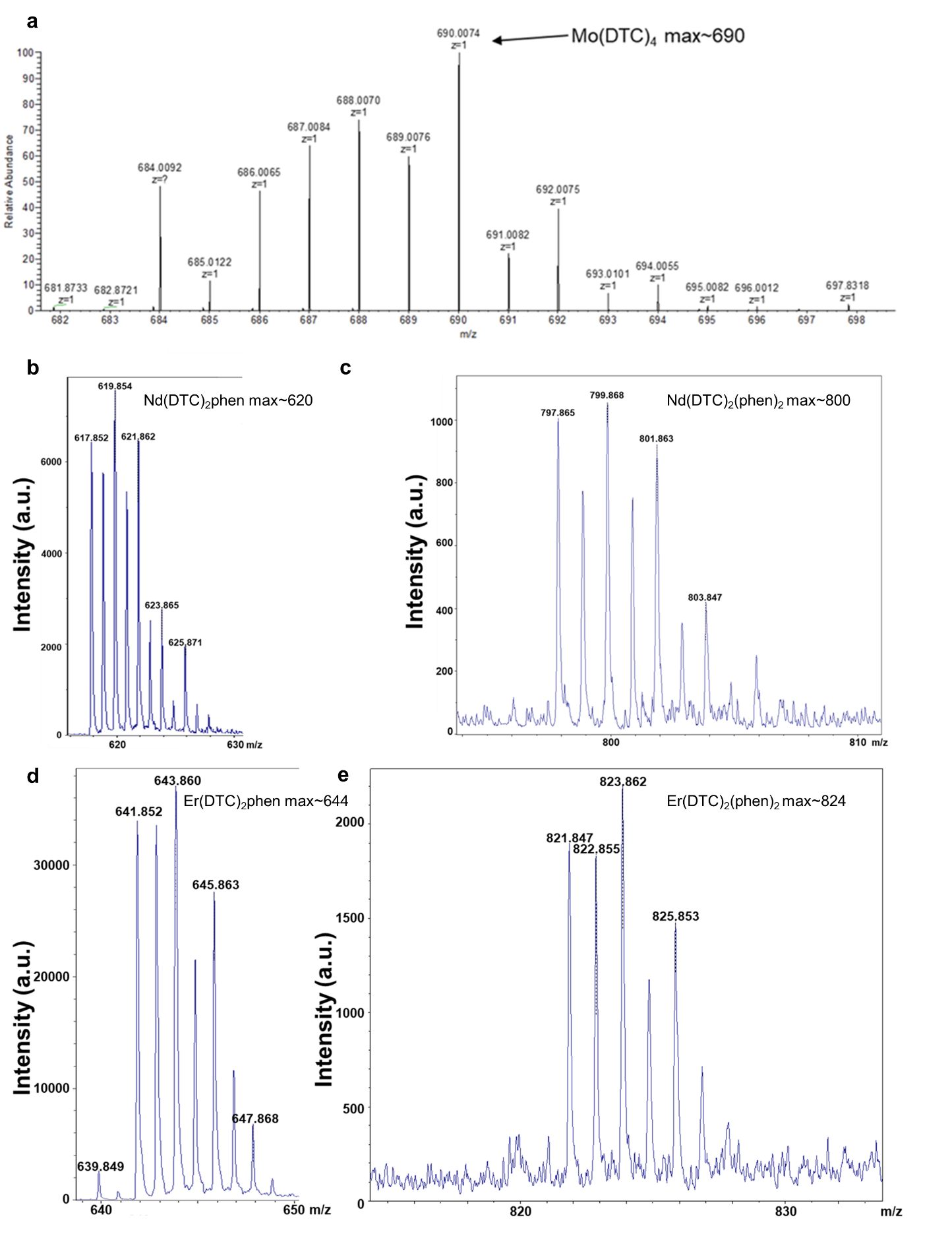
Figure S1. Mass spectrum of fabricated precursors with relatively strong intensity in selected regions for corresponding fragments **a**, Mo(DTC)_4_, **b**, Nd(DTC)_2_Phen, **c**, Nd(DTC)_2_(Phen)_2_, **d**, Er(DTC)_2_Phen and **e**, Er(DTC)_2_(Phen)_2_. The fragment patterns were identified via Chemputer.

Table S1. The CHN composition measurement result and theoretical percentages for synthesized Mo-, Nd-, and Er-precursors.

| Composition  Sample | | C (%) | H (%) | N (%) |
| --- | --- | --- | --- | --- |
| Mo(DTC)_4_ | Measured | 34.55 | 5.90 | 7.69 |
|  | Theoretical | 34.87 | 5.85 | 8.13 |
| Nd(DTC)_3_Phen | Measured | 41.80 | 4.91 | 9.04 |
|  | Theoretical | 42.16 | 4.98 | 9.10 |
| Er(DTC)_3_Phen | Measured | 39.58 | 4.91 | 8.61 |
|  | Theoretical | 40.93 | 4.83 | 8.84 |

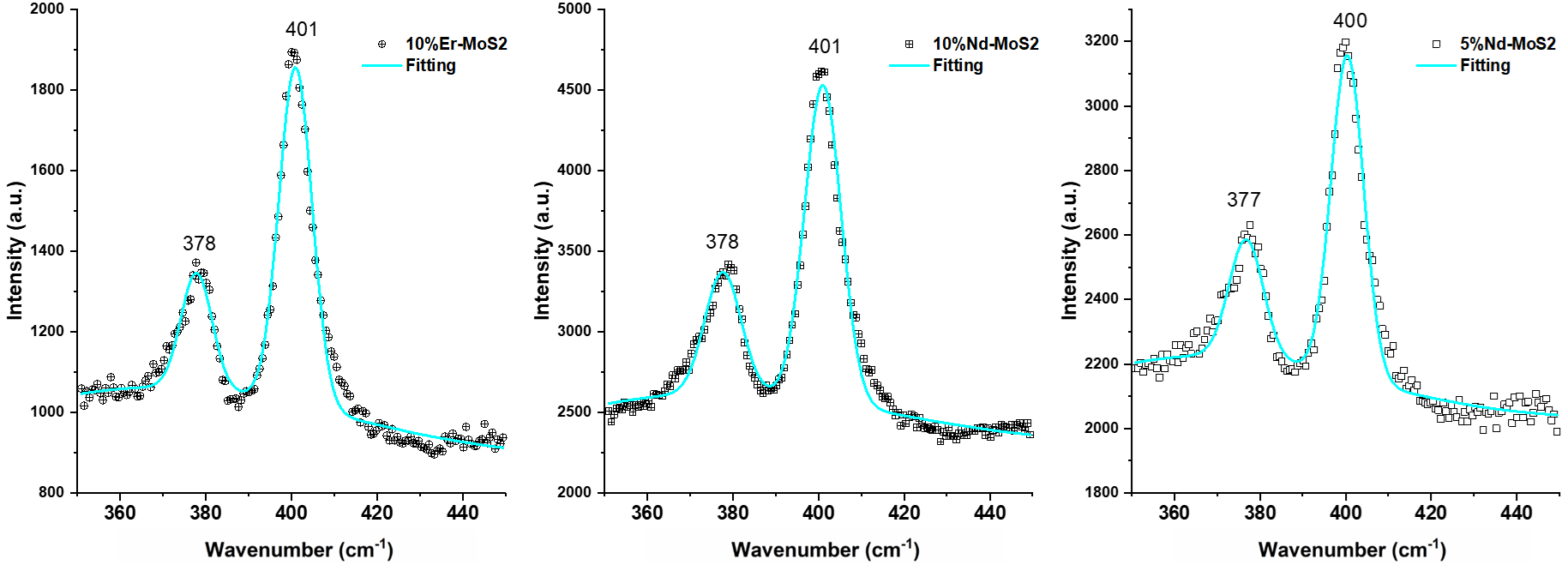
Figure S2. An example of fitting the XRD data of 5%-Er MoS_2_ (top) with the estimated position and FWHM of (100) and (110) peaks and subtracted background. The baseline was established using a spline fixed by original data points at 28°, 54° and 65°, which results in non-zero values at boundaries (10° and 80°) after background subtraction; Raman shift spectra of 10% Er doped (bottom left), 10% Nd doped (bottom middle), and 5% Nd doped MoS_2_ (bottom right) in range 350 ~ 450 cm^-1^ and excited by a 488 nm laser.

| Index |  | Undoped | 5%Er | 5% Nd | 10%Er | 10%Nd |
| --- | --- | --- | --- | --- | --- | --- |
| 100 | **Peak centre** | **33.7°** | **33.7°** | **33.7°** | **33.7°** | **33.8°** |
| 100 | **Peak width** | **2.4°** | **2.4°** | **2.5°** | **2.5°** | **3.0°** |
| 110 | **Peak centre** | **59.2°** | **59.2°** | **59.2°** | **59.2°** | **59.1°** |
| 110 | **Peak width** | **3.2°** | **3.3°** | **3.5°** | **3.4°** | **3.4°** |

Table S2. X-ray diffraction peak positions and widths for the doped and undoped samples. The precision of these measurements was determined by measuring the observed linewidth (full width half maximum) of a silicon standard.

Table S3. Raman peak positions and widths for the doped and undoped samples.

| Raman mode |  | Undoped  (cm^-1^) | 5%Er  (cm^-1^) | 5% Nd  (cm^-1^) | 10%Er  (cm^-1^) | 10%Nd  (cm^-1^) |
| --- | --- | --- | --- | --- | --- | --- |
| E^1^_2g_ | **Peak centre** | **378.0** | **377.8** | **376.8** | **377.9** | **377.7** |
| E^1^_2g_ | **Peak width** | **8.9** | **8.5** | **9.4** | **8.5** | **9.9** |
| A_1g_ | **Peak centre** | **401.0** | **400.9** | **400.5** | **401.0** | **401.0** |
| A_1g_ | **Peak width** | **8.6** | **8.7** | **9.0** | **9.1** | **10.0** |
| A_1g_ -E^1^_2g_ | **Difference in peak centres** | **23.0** | **23.1** | **23.7** | **23.1** | **23.3** |

Table S4. Relative atomic abundances, RAA, of Mo and Ln in each sample determined from EDX, and the doping fraction, x= RAA(Ln)/(RAA(Mo)+RAA(Ln)).

| RAA/ Doping Fraction | 5%Er | 5% Nd | 10%Er | 10%Nd |
| --- | --- | --- | --- | --- |
| Mo | **12.5** | **10.8** | **10.6** | **13.1** |
| Ln | **0.6** | **0.6** | **0.9** | **1.5** |
| x | **4.6%** | **5.2%** | **7.8%** | **10.3%** |


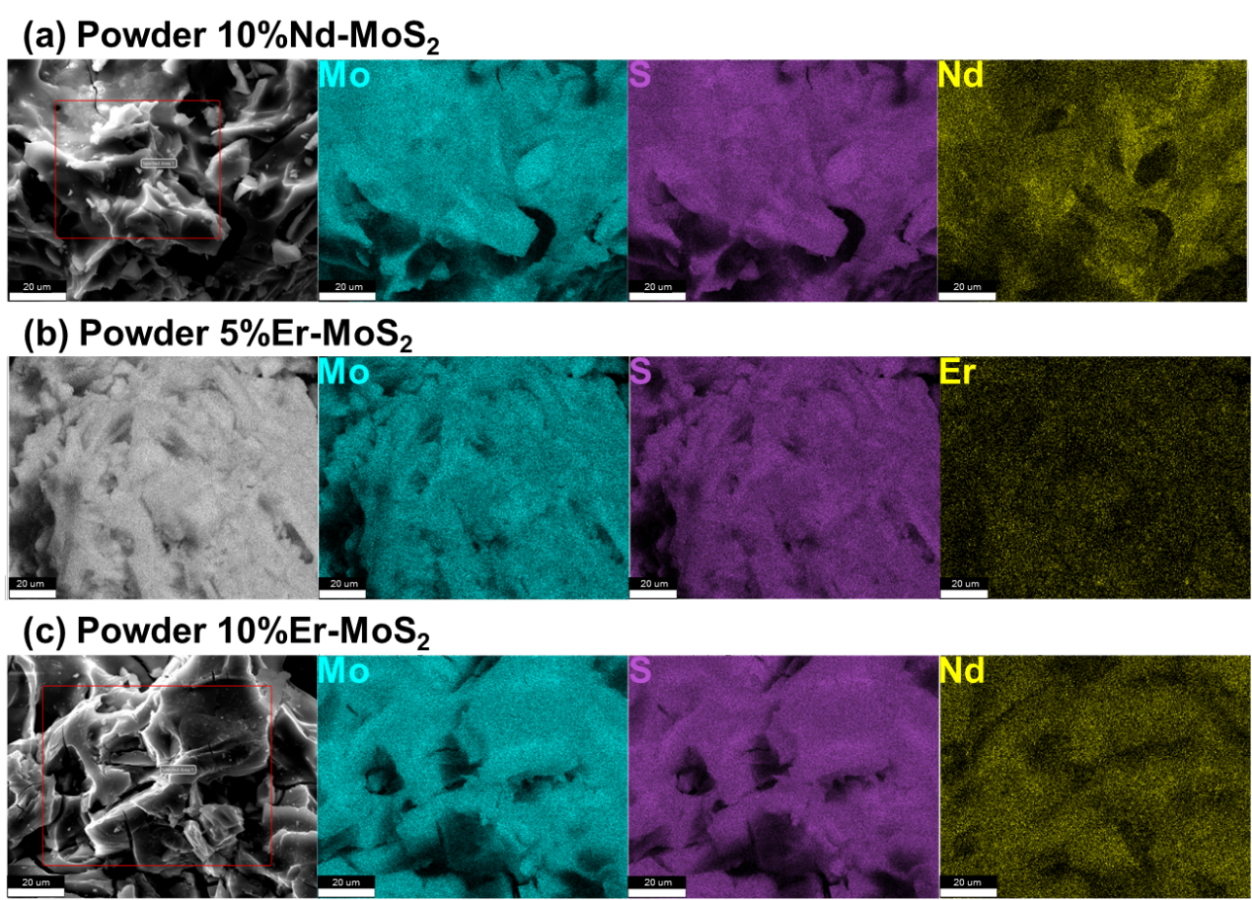
Figure S3. SEM/EDX elementary mapping of (a) 10% Nd doped, (b) 10% Er doped, (c) 5% Nd doped MoS_2_ powder sample with Mo, S, Er and Nd distribution.

Table S5. Variables used in fitting the 10% Nd-doped MoS_2_ XAFS data to the intralayer incorporation model. *N* is the coordination number of the Nd atom; *S_o_^2^* is the XAFS amplitude reduction factor, which is typically 0.90 and 0.91 for Nd and Er respectively ^1-3^; *σ^2^* is mean-square displacement; *ΔE_0_* (eV) is the shift from initial photo-electron energy *E_0_*; *Δr* is shift in path length; *R_eff_* is nominal path length, given by the structure model; *R = R_eff_* + *Δr*. Background removal parameter Rbkg = 1.2. The parameters *N* and *S_o_^2^* were set as constants. The *E_0_* was set 6208.00 eV, which is around the first derivative maximum of absorption spectrum. (n.b. all paths considered are single scattering)

| Shell | *N* | *S_o_^2^* | *σ^2^* (Å^2^) | *ΔE_0_* (eV) | *Δr* (Å) | *R_eff_* (Å) | *R* (Å) |
| --- | --- | --- | --- | --- | --- | --- | --- |
| Nd to nearest S | 6* | 0.90* | 0.012 ± 0.006 | -4.88 ± 5.38 | 0.26 ± 0.07 | 2.42 | 2.68 ± 0.07 |
| Nd to nearest Mo | 6* | 0.90* | 0.023 ± 0.035 | -4.88 ± 5.38 | 0.11 ± 0.22 | 3.19 | 3.30 ± 0.22 |

* The variable was fixed for EXAFS fitting, based on known structure and literature source.


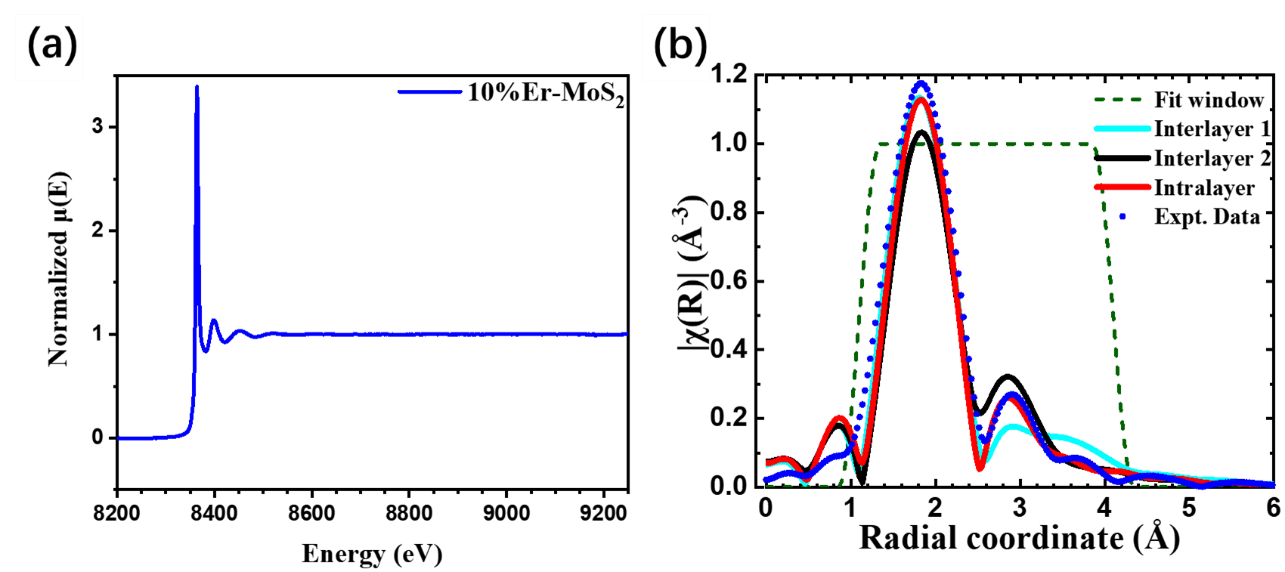
Figure S4. (a) The normalized absorption spectrum *μ*(*E*) for the 10% Er-doped MoS_2_ sample and (b) Fourier transform of the absorption spectrum and fitting in *R* space (without phase correction), with *k* weight of 2. Fourier transform is within 2.0 – 6.8 Å^−1^ in *k*-space applying a Hanning window function rolled off over 1 Å^−1^. Fitting over *R,* from 1.1 to 4.1 Å with a Hanning window rolled off over 0.5 Å, is compared for the interlayer model and both intralayer models.

Table S6. Variables used in fitting the 10% Er-doped MoS_2_ XAFS data to the intralayer incorporation model. Rbkg = 1.1; The parameter *N* and *S_o_^2^* were set as constant. The *E_0_* was set 8361.83 eV, which is around the first derivative maximum of absorption spectrum. See caption for Table S2 for parameter definitions. (n.b. all paths considered are single scattering)

| Shell | *N* | *S_o_^2^* | *σ^2^* (Å^2^) | *ΔE_0_* (eV) | *Δr* (Å) | *R_eff_* (Å) | *R* (Å) |
| --- | --- | --- | --- | --- | --- | --- | --- |
| Er to nearest S | 6* | 0.91* | 0.008 ± 0.004 | -9.84 ± 4.22 | 0.09 ± 0.05 | 2.42 | 2.51 ± 0.05 |
| Er to nearest Mo | 6* | 0.91* | 0.017 ± 0.021 | -9.84 ± 4.22 | -0.16 ± 0.15 | 3.19 | 3.03 ± 0.15 |

* The variable was fixed for EXAFS fitting, based on known structure and literature source.


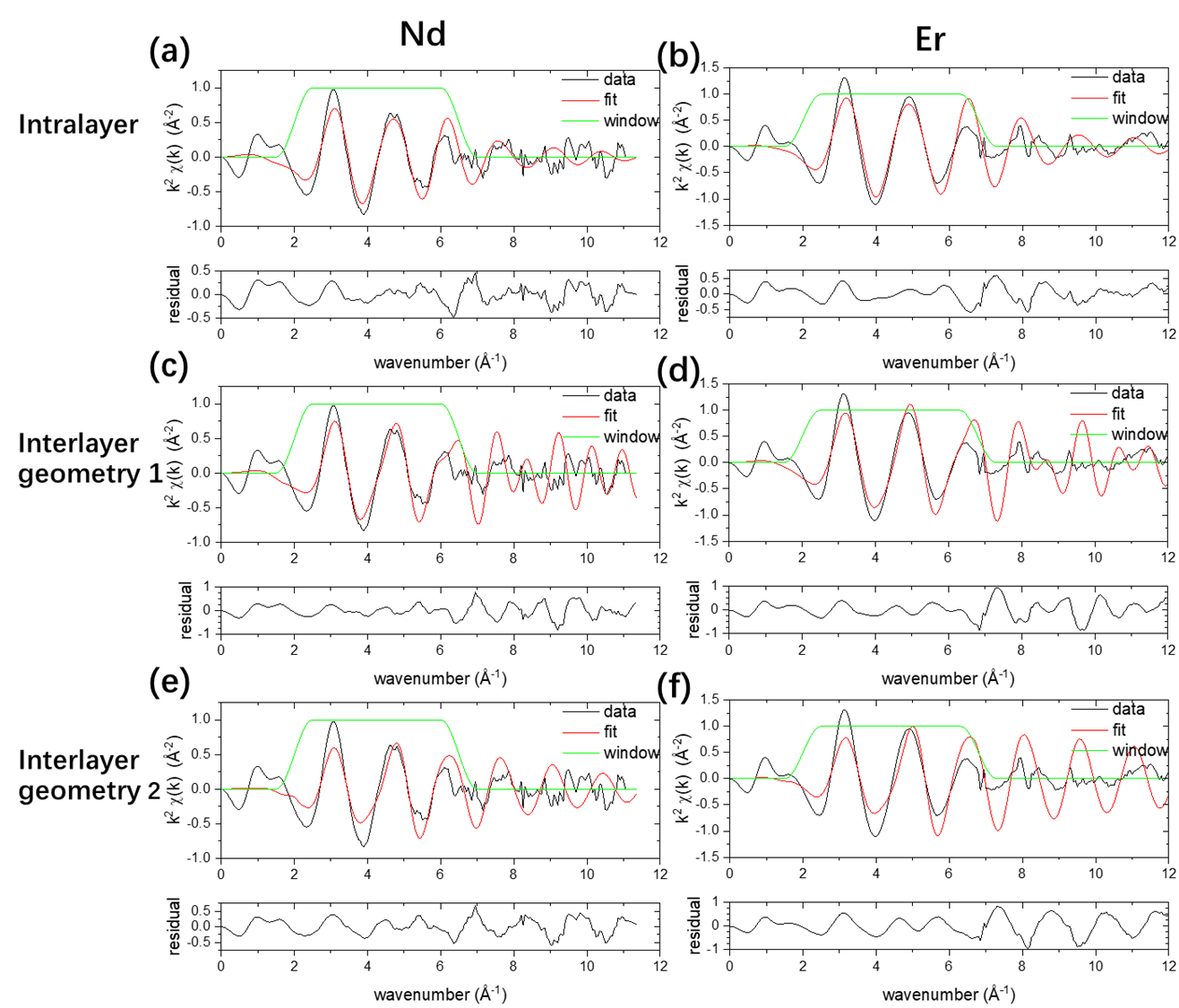


Figure S5. XAFS data in k-space with fits and residuals: to the intralayer model for (a) Nd-MoS_2_ and (b) Er-doped MoS_2_; to the interlayer model geometry 1 for (c) Nd-MoS_2_ and (d) Er-MoS_2_; and to the interlayer model geometry 2 for (e) Nd-MoS_2_ and (f) Er-MoS_2_ model.

Table S7. Variables used in fitting the 10% Nd- and 10% Er-doped MoS_2_ XAFS data for interlayer incorporation (geometry 1). Rbkg and *E_0_* were set up as the same as above. (n.b. all paths considered are single scattering)

| Shell | *N* | *S_o_^2^* | *σ^2^* (Å^2^) | *ΔE_0_* (eV) | *Δr* (Å) | *R_eff_* (Å) | *R* (Å) |
| --- | --- | --- | --- | --- | --- | --- | --- |
| Nd to nearest S | 6* | 0.90* | 0.011 ± 0.005 | -9.32 ± 5.64 | -0.19 ± 0.07 | 2.83 | 2.64 ± 0.07 |
| Nd to nearest Mo | 6* | 0.90* | 0.005 ± 0.018 | -9.32 ± 5.64 | -0.17 ± 0.18 | 4.15 | 3.98 ± 0.18 |

| Shell | *N* | *S_o_^2^* | *σ^2^* (Å^2^) | *ΔE_0_* (eV) | *Δr* (Å) | *R_eff_* (Å) | *R* (Å) |
| --- | --- | --- | --- | --- | --- | --- | --- |
| Er to nearest S | 6* | 0.91* | 0.006 ± 0.003 | -13.41 ± 4.41 | -0.35 ± 0.05 | 2.83 | 2.48 ± 0.05 |
| Er to nearest Mo | 6* | 0.91* | 0.005 ± 0.014 | -13.41 ± 4.41 | -0.38 ± 0.13 | 4.15 | 3.77 ± 0.13 |

* The variable was fixed for EXAFS fitting, based on known structure and literature source.


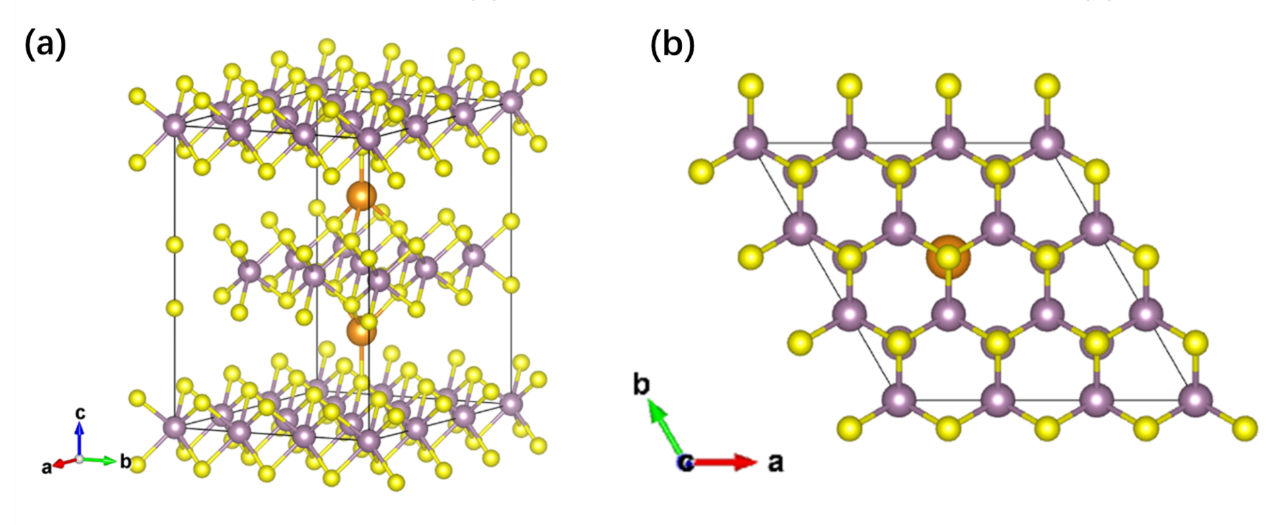
Figure S6. a) and b) Visualizations of the interlayer incorporation model geometry 2 (tetrahedron of nearest S). (n.b. the yellow, purple and orange balls represent S, Mo and RE atoms respectively).

Table S8. Variables used in fitting the 10% Nd- and 10% Er-doped MoS_2_ XAFS data for interlayer incorporation (geometry 2). Rbkg and *E_0_* were set up the same as above. (n.b. all paths considered are single scattering)

| Shell | *N* | *S_o_^2^* | *σ^2^* (Å^2^) | *ΔE_0_* (eV) | *Δr* (Å) | *R_eff_* (Å) | *R* (Å) |
| --- | --- | --- | --- | --- | --- | --- | --- |
| Nd to nearest S | 4* | 0.90* | 0.005 ± 0.007 | -6.27 ± 7.70 | 0.12 ± 0.09 | 2.55 | 2.66 ± 0.09 |
| Nd to second nearest S | 9* | 0.90* | 0.019 ± 0.034 | -6.27 ± 7.70 | 0.29 ± 0.28 | 4.08 | 4.38 ± 0.28 |

| Shell | *N* | *S_o_^2^* | *σ^2^* (Å^2^) | *ΔE_0_* (eV) | *Δr* (Å) | *R_eff_* (Å) | *R* (Å) |
| --- | --- | --- | --- | --- | --- | --- | --- |
| Er to nearest S | 4* | 0.91* | 0.001 ± 0.005 | -10.99 ± 6.29 | -0.06 ± 0.07 | 2.55 | 2.49 ± 0.07 |
| Er to second nearest S | 9* | 0.91* | 0.014 ± 0.022 | -10.99 ± 6.29 | 0.03 ± 0.19 | 4.08 | 4.12 ± 0.19 |

* The variable was fixed for EXAFS fitting, based on known structure and literature source.



Figure S7. The photoluminescence spectra of 5% Er doped MoS_2_ and reference at 14 K, excited by 400 nm laser. Possible Er transitions were labelled.


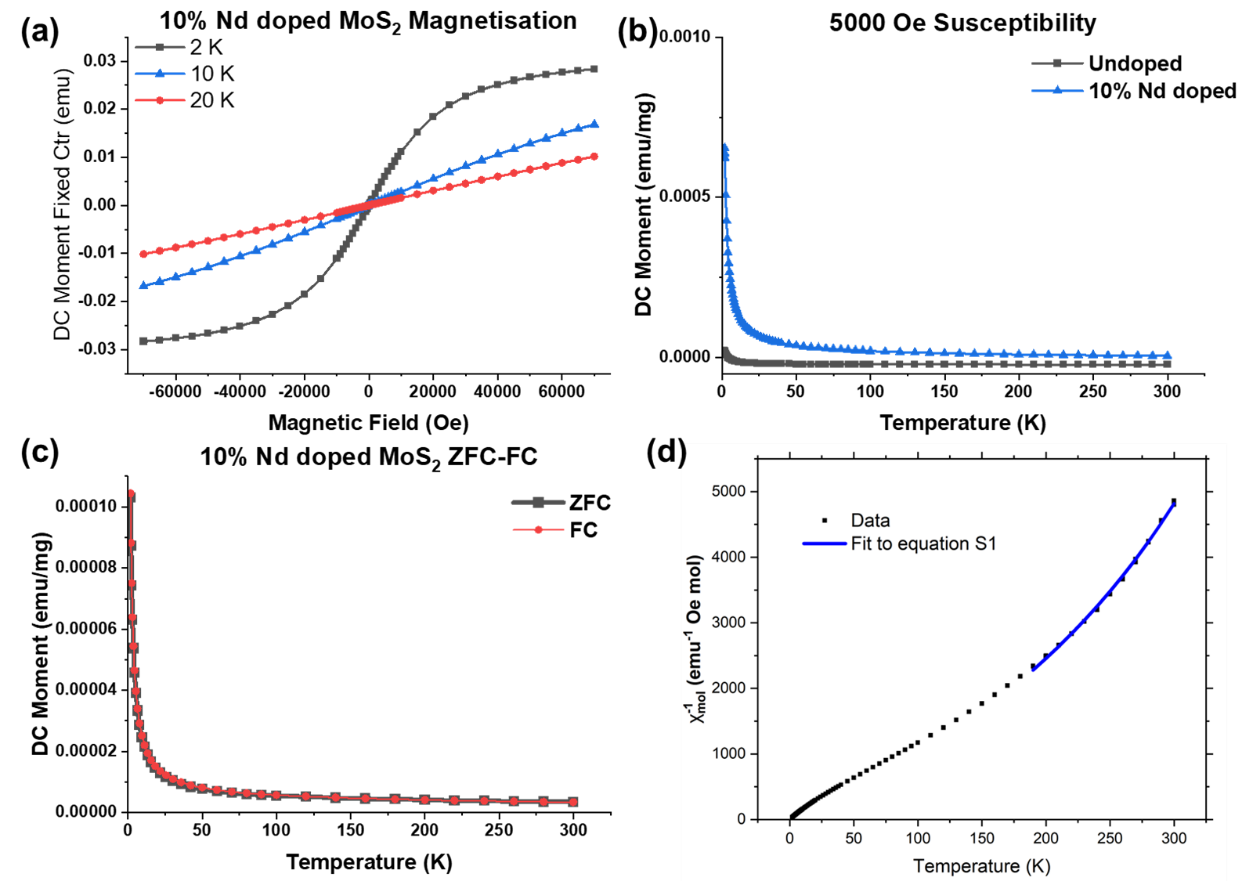


Figure S8. **a**, Magnetic response of the 10% Nd doped MoS_2_ sample at low temperature. **b**, Comparison of the temperature dependence of magnetization for 10% Nd doped and undoped MoS_2_ at 5000 Oe. **c**, Field cooled (FC) and zero field cooled (ZFC) magnetization response at 100 Oe for the 10% Nd-doped sample. d) Temperature dependence of the inverse molar susceptibility, $\chi_{mol}^{-1}$; the fit shown is to the Curie-Weiss law for temperature >200K with a temperature-independent contribution to susceptibility. ^4^

For samples with a temperature independent component to their molar susceptibility, $\chi_{0}$, the Curie Weiss Law describing the dependence of the molar susceptibility, $\chi_{mol}$ , on temperature, $T$ , is modified to ^4^:

$$\chi_{mol}^{-1}=\frac{T-T_{CW}}{\chi_{0}\left( T-T_{CW} \right)+C}$$

(S1)

where $C$ is the Curie constant and $T_{CW}$ is the Curie-Weiss temperature.

**References**

1. Y. Mao, J. Bargar, M. Toney and J. P. Chang, *Journal of Applied Physics*, 2008, **103**,  094316.

2. G. Mountjoy, J. M. Cole, T. Brennan, R. J. Newport, G. A. Saunders and G. W. Wallidge, *Journal of Non-Crystalline Solids*, 2001, **279**, 20-27.

3. T. T. Van, J. R. Bargar and J. P. Chang, *Journal of Applied Physics*, 2006, **100**, 023115.

4. S. Mugiraneza and A. M. Hallas, *Communications Physics*, 2022, **5**, 95.
