# Supplementary material for: Synthesis of rare earth doped MoS2 by the co-pyrolysis of molecular precursors
Source: Sci Rep. 2026 Mar 19;16:14252. doi: 10.1038/s41598-026-44301-1 (PMC13139367; doi:10.1038/s41598-026-44301-1)
Supplement: Supplementary file 2 — Supplementary Material 2 [file 41598_2026_44301_MOESM2_ESM.docx]

**Supporting Information**.

Mass spectra for the Mo, Er and Nd precursors; CHN compositional analysis for the Mo, Er and Nd precursors; XRD data fitting of 5% Er-doped MoS_2_ powders (example); Raman spectra for the 10% doped Er- and 5% and 10% Nd-doped MoS_2_ powders; SEM images and EDX maps for the 5% and 10% doped Er- and 10% Nd-doped MoS_2_ powders; XAFS data for the 10% Nd- and 10% Er-doped samples; PL spectrum for the 5% Er-doped MoS_2_ powder; Magnetic response for the 10% Nd-doped MoS_2_ sample.
